# The Migration-Mortality Hypothesis Requires Reproductive Compensation Not in Evidence

**DOI:** 10.3390/insects17101089

**Published:** 2026-10-21

**Authors:** Wayne E. Thogmartin

**Affiliations:** Upper Midwest Environmental Sciences Center, U.S. Geological Survey, 2630 Fanta Reed Road, La Crosse, WI 54603, USA; wthogmartin@usgs.gov

**Keywords:** monarch butterfly, *Danaus plexippus*, population decline, migration mortality, overwintering population, population dynamics

## Abstract

The migration-mortality hypothesis (also called the migration limitation hypothesis) attributes the decline in the eastern North American monarch butterfly (*Danaus plexippus*) overwintering population in Mexico to mortality incurred during autumn migration, while treating summer breeding populations in the United States and Canada as comparatively stable. A number of studies have raised empirical concerns about this stability premise itself, but here I set that debate aside and ask a narrower, conditional question: even if summer breeding abundance were as stable as the hypothesis requires, is the hypothesis internally coherent as a population-dynamics claim? I show, using a simple population-recovery framework, that the hypothesis carries a previously unstated and unexamined requirement: spring reproduction in the first generation must compensate for fall/winter losses in near-exact proportion, year after year, to reconcile a stable summer count with a declining winter count. I illustrate this requirement against summer (North American Butterfly Association, NABA) and winter (overwintering colony area) survey data from 2006 to 2015, the same period in which the hypothesis was first formulated and the same data used to motivate it. Required compensatory increases in spring reproduction needed to generate a stationary summer population often exceeded 250%, including an increase of 930% (a 10.3-fold increase) in 2013. No published evidence supports a compensatory increase in spring reproduction of the magnitude or consistency the hypothesis demands. I argue this omission is a logical structural weakness of the migration-mortality hypothesis, independent of, and in addition to, the sampling and interpretation concerns already raised in the literature, and I outline what data would be needed to test the compensatory mechanism directly (e.g., spring reproductive output). Until such data exist, invoking migration mortality as an explanation for the discrepancy between summer and winter counts asks readers to accept a compensatory mechanism with no evidentiary basis, a standard the hypothesis’ proponents have not held themselves to, and one the field should no longer accept.

## Simple Summary

Scientists studying the monarch butterfly have long debated why the number of butterflies counted in their winter colonies in Mexico has gone down while counts made during the summer in the United States and Canada do not show the same clear drop. One idea proposes that most losses occur during the long fall journey south, but this study shows that this explanation requires an unlikely pattern: every spring, the butterfly population would need to grow by just the right amount to fully replace the missing butterflies from the previous fall and winter. Examining data from 2006 to 2015, this study found that this required level of spring growth is often extremely large, far greater than anything supported by current knowledge of butterfly biology. In one year, the required increase was more than ten times the number of butterflies counted at winter colonies. No evidence has been presented that such large and precise increases actually occur. This means the migration-related explanation is incomplete and needs direct testing with new data. Understanding the true causes behind monarch declines is essential for choosing effective conservation actions that protect this well-known and widely appreciated species.

## 1. Introduction

The eastern North American population of the monarch butterfly (*Danaus plexippus*) declined substantially in its Mexican overwintering grounds over the past three decades, as measured by the area of forest occupied by overwintering colonies [1]. The cause of this decline, for many, remains contested. The threats are multifarious [2,3], but leading explanations include: (1) the milkweed limitation hypothesis, which attributes the decline to loss of milkweed (*Asclepias* spp.) host plants in the breeding range following the adoption of glyphosate-tolerant crops in the U.S. Midwest [4,5]; (2) habitat degradation at overwintering sites in Mexico [6]; and (3) the migration-mortality hypothesis (also called the migration limitation hypothesis), which holds that summer breeding-ground populations have not meaningfully declined, and that the observed decline in overwintering colony size instead reflects rising mortality occurring during the roughly 3000 km fall migration itself, prior to colony surveys in Mexico [7].

The migration-mortality hypothesis developed across a series of publications beginning in the early 2010s [8,9,10,11,12] (originating with Badgett and Davis [8]). The hypothesis arose as a direct counterpoint to milkweed limitation: researchers observed that summer breeding-ground population sizes in the Midwest did not appear to decline as steeply as the overwintering colony area in Mexico, if at all, suggesting a “disconnect” between the two life stages that milkweed loss alone could not explain. Badgett and Davis [8] explicitly hypothesized that diminished fall migration success, rather than reduced summer breeding output, was an important driver of the overwintering decline. Davis and Dyer [9] examined nectar and energetic resource constraints along the migratory corridor as a mechanistic basis for migration-related mortality. A related 2016 analysis [10] largely rejected milkweed limitation as an explanation for summer breeding-season dynamics, pointing instead toward problems during the fall transition, positing sparse autumnal nectar sources, adverse weather, and habitat fragmentation along the corridor as the source of increased mortality, though notably with no data to support that supposition.

The fall migration has been framed as a Darwinian “selective episode” capable of removing a substantial fraction of the summer-produced population before it reaches Mexico [11], and research has continued to pursue empirical support for the hypothesis. A 2024 analysis of citizen-science roost-count data reported progressive, distance-dependent declines in migratory roost sizes along the flyway, up to 80% at the southernmost portions of the route, even as climate conditions should, on other grounds, have favored the population [12]; their proposed mechanisms for migration-stage mortality include rising parasite loads (potentially linked to non-native milkweed and captive-reared “backyard” releases), habitat fragmentation, and reduced nectar availability along the corridor; importantly, Davis et al. [12] showed that monarch butterflies are more likely to die the further they travel but did not establish a pattern of increasing deterioration in migration survival over multiple years. Separately, long-term Cape May, New Jersey, monitoring data have been used to document a phenological shift in migration timing of more than two weeks since 1992, with late-migrating individuals recovered in Mexico at substantially lower rates, evidence marshaled in support of a migration-stage mechanism for the observed decline [13].

The migration-mortality hypothesis has not gone unchallenged [14,15]. For example, Taylor et al. [16] analyzed approximately 1.4 million tagged monarchs and 14,000 recoveries in Mexico, compiled by Monarch Watch, and found no decline over time in tagging recovery rates, a direct proxy for migration success, and instead found that the number of monarchs tagged each summer correlated strongly with the following winter’s overwintering population size, a pattern more consistent with summer-breeding (milkweed) limitation than with migration mortality. Fordyce et al. [17] challenged this interpretation, arguing that tag-recovery rate may not reliably reflect migration survival due to confounding factors such as observer effort, tagging-site distribution, and spatial variation in recovery reporting, and cautioned that a stable recovery rate does not necessarily rule out increasing migration mortality if effort-related biases shift in an equal but compensating direction over the same period; notably, though, there is no evidence or rationale supporting such compensatory shifts.

Further, large-scale climate-focused analyses by Zylstra et al. [18] reported evidence for population declines originating in the summer breeding population rather than during migration, contrasting with earlier analysis that had supported the migration-survival account [10]. Similarly, Diffendorfer et al. [19], using vegetation greenness (Normalized Difference Vegetation Index) as a proxy for nectar and habitat resource availability, found comparatively small effects of landscape and climate change on late-fall and spring migratory habitat, results inconsistent with the increasing deterioration in the fall migration required by the migration-mortality hypothesis. Additionally, Pleasants et al. [20,21] have separately raised methodological concerns about how breeding-ground surveys are interpreted, and directly compared summer, fall, and winter population estimates before and after milkweed loss, finding evidence inconsistent with a migration-mortality account.

My purpose here is complementary to this empirical debate rather than a re-adjudication of it. I set aside sampling and survey-interpretation concerns and ask a narrower question: is the migration-mortality hypothesis internally coherent as a population-dynamics claim, independent of any data-quality dispute? I argue that it is not, or at minimum, that it carries a crucial assumption that should be acknowledged and tested: that spring reproduction must compensate for fall and winter losses in almost exactly the amount needed to restore the population to its prior breeding-ground abundance in every year in which a loss occurs.

## 2. The Logical Structure of the Migration-Mortality Hypothesis

Consider the minimal set of claims the migration-mortality hypothesis requires:

1. Summer breeding populations (measured, e.g., by North American Butterfly Association counts or similar breeding-ground indices) are approximately stable over time.
2. Winter overwintering populations (measured by colony area in Mexico) are declining over time.
3. The discrepancy between (1) and (2) arises because of mortality occurring during fall migration or overwinter, prior to the point at which winter surveys are conducted.

Claims (1)–(3) are not, by themselves, contradictory. But they are incomplete. If fall/winter mortality increases over time, and yet summer populations the *following* year remain stable, something must be replacing the individuals lost (Figure 1). Under standard monarch life-history, where the population overwintering in Mexico is the same generation that begins the spring remigration and produces the first-generation offspring that repopulate the summer breeding range [22], the only mechanism available to close this gap is reproduction in the early spring generation, before summer surveys are conducted.

This outcome yields a fourth, implicit claim that the hypothesis requires but does not state:

4. Total reproductive output in spring must increase each year by an amount that offsets the fall/winter mortality increase from the preceding season—closely enough, and consistently enough, that summer populations show no resulting trend.

Claim (4) is a strong and specific quantitative prediction. It is worth being explicit about why this claim, and not simply the observation of a summer/winter discrepancy itself, is the analytical target of this paper. A stable summer count alongside a declining winter count is, on its face, merely a pattern requiring *some* explanation; it does not by itself specify a mechanism. The migration-mortality hypothesis is a specific candidate explanation, one that locates the entire discrepancy in post-breeding, pre-census mortality. Accepting that explanation, rather than treating the discrepancy as an open empirical question to be resolved by whichever mechanism the data eventually support, is what commits a proponent to claim (4): if summer stability is real and the shortfall is real, and the hypothesis attributes the shortfall entirely to migration-stage mortality rather than to a summer-stage process, then only spring reproductive compensation remains available to reconcile the two counts. The compensatory-reproduction requirement is therefore not an incidental feature of how the hypothesis happens to be stated; it is the arithmetic consequence of adopting the hypothesis’ own causal claim, and it is a consequence that has not, to my knowledge, been previously identified or tested.

Crucially, claim (4) requires that reproduction must scale compensatorily and repeatedly, tracking a moving target (the size of the preceding fall/winter loss) closely enough to erase its signature from the summer count in every year the discrepancy is observed. Below, I demonstrate that mathematically this repeated compensation was implausible at the time this hypothesis arose, during and shortly after the period when monarchs were declining according to winter surveys in Mexico.

## 3. A Simple Formalization

I do not myself take summer-population stability as established; Pleasants et al. [20,21] questioned whether breeding-ground surveys support this conclusion. Rather, I grant claim (1) arguendo, because it is the premise the migration-mortality hypothesis itself requires. My argument is conditional: even if summer stability holds exactly as the hypothesis needs it to, the hypothesis still requires an unevidenced compensatory mechanism.

To illustrate this unevidenced compensatory mechanism, I posit a simplified, deterministic model. Let *S*(*t*) denote the breeding-ground (summer) population index in year *t*, and *W*(*t*) denote the overwintering population index measured going into year *t*. Under the migration-mortality hypothesis:

*S*(*t*) ≈ *S*_0_ (*approximately constant*)

*W*(*t*) = *W*_0_ − *δ*(*t*) (*declining*, *with increasing loss δ*(*t*) *over time*)

For the population to return to *S*_0_ by the following summer, spring reproduction *R*(*t*) must satisfy, approximately,

*W*(*t*) + *R*(*t*) ≈ *S*_0_

*R*(*t*) ≈ *S*_0_ − *W*(*t*) = *δ*(*t*)

That is, required reproductive compensation *R*(*t*) must track the fall/winter deficit *δ*(*t*) almost exactly, year over year. If *δ*(*t*) is increasing over time, which it must be for winter populations to show a declining trend, then *R*(*t*) must also be increasing over time, at a matched rate, purely to hold *S*(*t*) constant.

This formalization is intentionally simple. It treats *S*_0_ as fixed and the relationship between loss and compensation as linear and exact, and it does not incorporate stochastic year-to-year variation in vital rates, density dependence, or measurement error in either survey. These simplifications are appropriate for the purpose at hand: the goal is to make explicit, in the clearest possible terms, an implicit logical requirement of the hypothesis as it is typically stated, not to build a general-purpose predictive model of monarch demography. A more elaborate stochastic treatment could be a natural extension for future work, particularly if paired with the empirical measurements outlined in Section 5, but is not needed to establish the structural point made here.

## 4. Illustrative Analysis

I examine the 2006–2015 period. This 2006–2015 window is not an arbitrary or dated convenience sample; it corresponds to the period during which the migration-mortality hypothesis was first articulated and empirically developed, beginning with Badgett and Davis [8] and Davis and Dyer [9], using summer and winter survey records from this same era (Figure 2). Using this same window allows a direct test of the hypothesis on its own founding evidence, rather than asking whether a later, independently collected dataset happens to reproduce the pattern. If the compensatory-reproduction requirement described in Section 3 cannot be supported using the very data that motivated the hypothesis, the burden falls on the proponents to demonstrate it in any subsequent period, not on critics to keep re-testing it against a moving dataset. I therefore treat 2006–2015 as the relevant evidentiary basis for this argument, and do not extend the empirical analysis beyond it here.

It is worth being explicit, however, about why I do not extend the quantitative analysis into 2016–2025, since more recent data now exist and a reader might reasonably expect them to be addressed. Since circa 2008, the eastern migratory population has been characterized by a mean growth rate near replacement (λ ≈ 1) rather than the sustained decline that characterized the earlier period [21,23]. Because the compensatory-reproduction signature this paper investigates is only meaningful, and only detectable, when a substantial and *growing* deficit δ(t) exists between summer and winter counts, a period in which that deficit is largely absent cannot serve as a test of claim (4); there is little or no compensatory burden to detect in the first place. Extending the regression analysis into this later period would not strengthen or weaken the argument made here, it would simply answer a different question, about a different demographic regime, in which the mechanism under scrutiny is not obviously called upon at all.

Standardized summer (NABA) and winter (aligned overwintering colony area, OW) population indices were regressed against year (2006–2015) using ordinary least squares to test for linear trend in each series independently (see the Supplementary Materials for all data and code). A third regression tested for linear trend in the annual deficit between the two series, calculated as the signed difference (summer minus winter, both standardized, with OW aligned to NABA’s 2006 starting value). This deficit represents the compensatory reproductive signal that the migration-mortality hypothesis requires, per the formalization in Section 3. All three regressions were fit with year as the sole predictor (*n* = 10).

Annual population data of this kind may exhibit a temporal autocorrelation, which ordinary least squares does not account for and which, if present, could understate the uncertainty in the reported trend estimates. The sample size for each regression is, however, small, hampering reliable accommodation of autocorrelation; the small sample size also limits statistical power, particularly for the deficit-trend regression. The purpose of the regressions, though, is meant to be illustrative, to characterize the qualitative shape of each series over the founding period of the hypothesis, rather than to produce definitive inferential tests. With these caveats in mind, the summer (NABA) trend was not statistically distinguishable from zero (b = −0.098/year, R^2^ = 0.088, *p* = 0.404) (Figure 2), consistent with the assumption of stability in breeding-ground counts that the migration-mortality hypothesis requires. The winter (OW) trend showed a statistically significant decline over the same period (b = −0.226/year, R^2^ = 0.469, *p* = 0.029) (Figure 2), confirming that overwintering colony area declined reliably across this window.

I then expect that the deficit between summer and winter, the quantity that spring reproductive compensation would need to track under the migration-mortality hypothesis, should increase over time, and increase in a manner traceable to an independently documented reproductive mechanism. The deficit between the two series showed a positive point estimate that did not reach conventional statistical significance (b = +0.128/year, R^2^ = 0.304, *p* = 0.098) (Figure 3). This absence of a coherent deficit trend is inconsistent with the sustained, scaling compensatory mechanism that the migration-mortality hypothesis requires; it does not demonstrate that compensation never occurs, but it indicates that only a weak signature of it is present in the data that motivated the hypothesis.

Given the observed year-over-year decline in winter colony area, what percentage increase in spring reproductive output would be required to restore summer counts to their prior level? To place the deficit trend in a biologically interpretable context, I calculated, for each year in the 2006–2015 series, the reproductive compensation that would be required in spring to restore the observed overwintering population (OW, hectares) to its 2006 baseline value. This value was expressed both as a multiplier (baseline value divided by the observed value in a given year) and as an equivalent percent increase over the observed value. This calculation was performed using OW in its original units, avoiding any dispute to the hectares-to-butterflies conversion factor [24] and any presumed equivalence between OW and NABA units. I note, however, that this approach carries its own assumption: it treats colony area as linearly proportional to total overwintering abundance [24]. If butterfly density per hectare varies systematically with colony size or year, for example, if smaller, more recent colonies are less densely packed than the larger colonies observed early in the series, then the required compensation would be biased low, i.e., the compensation necessary to overcome the deficit would be heightened.

Required compensatory increases in spring reproduction varied substantially across the study period (Figure 4), ranging from near parity in years with minimal winter decline (+36–71%) to a required increase of 930% (a 10.3-fold increase) in 2013, the year of lowest recorded overwintering colony area. A 258% increase in a single generation (as in 2009) requires that each female’s egg output must convert into roughly 4+ surviving adult female offspring, whereas increases of ~500% (as in 2012 and 2014) require 6+ surviving adult female offspring. A 930% increase requires 10+ surviving adult female offspring at the conclusion of this first spring reproduction.

For context, I also calculated the year-over-year percent change in OW itself across 2006–2015, independent of any fixed baseline. These changes were substantial and highly variable, ranging from a 62.1% decline (2008–2009) to a 254.9% increase (2014–2015), with a mean absolute year-over-year change of 74.3%. This year-to-year variability provides a natural benchmark against which to judge the magnitude of compensation the migration-mortality hypothesis requires: even relative to the winter population’s own considerable natural volatility, the reproductive compensation required in 2013 (+930%) is roughly 12.5 times the average year-over-year fluctuation observed anywhere in the winter series, and substantially exceeds even its single largest observed swing.

As a separate plausibility check, I consider an approximate upper bound on single-generation reproductive compensation based on maximum female fecundity. Oberhauser [25] reported an estimated average lifetime fecundity of approximately 410 eggs per female. Field studies indicate that monarch survival from egg to eclosing adult is typically low, often approximately 1–5% under unprotected conditions, with substantial variation attributable to site conditions, seasonal timing, weather, and natural enemies [26,27,28]. At the upper end, then, 5% survival would equate to approximately 10 adult female monarchs produced by *every* spring migrating adult female. Oberhauser et al. [22] found that increases of 1–2% in relevant vital-rate parameters, sustained across the full annual cycle and multiple regions, were necessary to move the modeled population from a mean declining trajectory (λ = 0.957) to a mean stable or growing one (λ ≥ 1). The compensation required to offset the 2013 winter deficit within a single spring generation therefore exceeds, by roughly two orders of magnitude, the scale of sustained structural improvement that published demographic modeling treats as an ambitious, internationally coordinated conservation target. This disparity was not limited to 2013: multiple years in the series required compensatory increases well beyond both benchmarks, the population’s own natural volatility and the vital-rate improvements demographic modeling treats as attainable. Thus, the migration-mortality hypothesis’ implicit reproductive-compensation requirement is not merely undocumented but, in several years, appears to exceed plausible biological capacity as characterized by existing demographic data and by the system’s own observed dynamics.

Beyond the implausibility of the compensatory mechanism, the migration-mortality hypothesis implicitly requires that migration-stage mortality intensified year-over-year during the period of population decline and then abated as the population stabilized. No such mechanism has been independently documented in the literature. This unstated assumption compounds the logical difficulties of the hypothesis and underscores the need for direct empirical testing of the compensatory requirements.

## 5. What Data Would Resolve the Compensation Question?

I am aware of at least one long-running, publicly available dataset with potential relevance to claim (4): the Monarch Larva Monitoring Program (MLMP) has collected weekly, state-level counts of milkweed plants monitored alongside egg, larval, and pupal counts in the area where the first generation arises (Texas and surrounding states) since the early 2000s, including the 2006–2015 period examined here [29,30,31]. In principle, these data could be aggregated into a milkweed-adjusted annual index of reproductive activity (e.g., eggs and larvae per milkweed plant monitored) to test for an increasing trend over this period. However, this index would not directly estimate *R*(*t*) as claim (4) requires. MLMP participation and monitoring effort have grown over time in ways not fully captured by milkweed-plant counts alone, and a per-plant reproductive index does not by itself establish total landscape-level reproductive output, nor does it extend past pupation to confirm survival into the reproducing adult population that would need to augment summer counts. I am not aware of a published analysis that overcomes these limitations to directly estimate first-generation spring reproductive output, adjusted for effort, over a time series long enough to test claim (4). Doing so would require data addressing three distinct components of the claim, each necessary but individually insufficient.

First, as noted, a direct measure of spring reproductive output is needed for the narrow window in which the returning overwintered generation reproduces, tracked over years spanning both rising and falling *δ*(*t*). Without contrast across years of differing fall/winter loss, no dataset, however precise, could establish whether reproductive output tracks the deficit as claim (4) requires.

Second, under standard monarch life history, overwintered adults reproduce in the southern spring range and then die; they do not persist alongside their offspring into the summer breeding population [22]. As a result, the relevant test is not one of distinguishing surviving originals from new recruits within a single summer census; nearly the entire subsequent breeding-range population consists of first-generation offspring by default. The more direct empirical target is therefore a sequential-cohort comparison: an estimate of the returning overwintered adult population as it arrives and begins reproducing in the spring range (approximating *W*(*t*) in the notation of Section 3), followed by an estimate of first-generation adult abundance in that same area once the parent generation has senesced. The ratio of these two sequential counts would provide a direct, empirical estimate of the realized per capita reproductive multiplication achieved that spring, allowing direct comparison against the compensation magnitudes required under claim (4) (Section 4). This approach depends on survey timing and a design capable of resolving the two cohorts with minimal temporal overlap, which is a nontrivial but tractable methodological challenge.

Third, even with reproductive output and *R*(*t*) both estimated directly, testing claim (4) requires a model connecting fall/winter mortality to spring reproduction across time—propagating estimated losses forward to predicted summer abundance, rather than comparing summer and winter counts as independent, post hoc quantities. Multi-generational demographic modeling of this kind (e.g., in the tradition of [4,22]) provides a framework capable of this integration, but has not, to my knowledge, been used explicitly to test the compensatory mechanism the migration-mortality hypothesis requires.

Some infrastructure relevant to the first component already exists. The Integrated Monarch Monitoring Program (IMMP) includes a standardized, geographically representative egg and larva survey protocol [31], and the Monarch Larva Monitoring Program (MLMP) has collected comparable data for decades, as noted above. However, published IMMP results to date have addressed only milkweed density and floral resource availability [32]; egg and larval data collected under the program have not yet been analyzed or reported, and existing sampling has been concentrated disproportionately outside the southern spring breeding range most relevant to this question [32]. Neither IMMP nor MLMP, to my knowledge, has been used to generate the sequential-cohort adult estimates described in the second component above. Moreover, IMMP data collection began in 2016, after the 2006–2015 period examined in Section 4; since approximately this time, and as noted in Section 4, the eastern migratory population has been characterized by a mean growth rate near replacement (λ ≈ 1), with continued year-to-year stochastic variation and non-negligible longer-term extinction risk [23,33], in contrast to the sustained decline that manifested into the 2006–2015 period. Because a compensatory-reproduction signature would only be expected to manifest when a substantial deficit δ(t) exists, current monitoring data, collected during a period without a comparable sustained deficit, cannot directly test claim (4) as it applied to the founding period of the hypothesis.

Absent this kind of evidence, the migration-mortality hypothesis is not nullified by this argument, but it is incomplete: it explains the discrepancy between summer and winter counts by asserting a compensatory mechanism whose existence and magnitude have not been recognized, described conceptually, or independently demonstrated.

## 6. Conclusions

The migration-mortality hypothesis, as described in the literature, is not self-contained. It implicitly requires that spring reproduction compensate almost exactly for fall and winter losses, in increasing measure, to reconcile stable summer counts with declining winter counts and, further, that whatever mechanism drives that increasing loss itself intensified across roughly 2006–2015 and subsequently abated, without independent documentation of either the compensatory mechanism or its reversal. A hypothesis that requires an undocumented mechanism to operate in exact, repeated proportion to an unrelated loss, and that fails to produce even the orderly trend its own logic demands, is not a competing explanation for the monarch’s decline. I suggest that future defenses of the migration-mortality hypothesis should directly address this mechanism, and I propose early-season reproductive surveys as the most direct means of doing so. Until such data exist, invoking migration mortality as an explanation for the discrepancy between summer and winter counts asks readers to accept a compensatory mechanism that currently lacks evidentiary support. Further evidence is therefore needed before this explanation can be evaluated and incorporated into the broader scientific consensus.

## Figures and Tables

**Figure 1:**
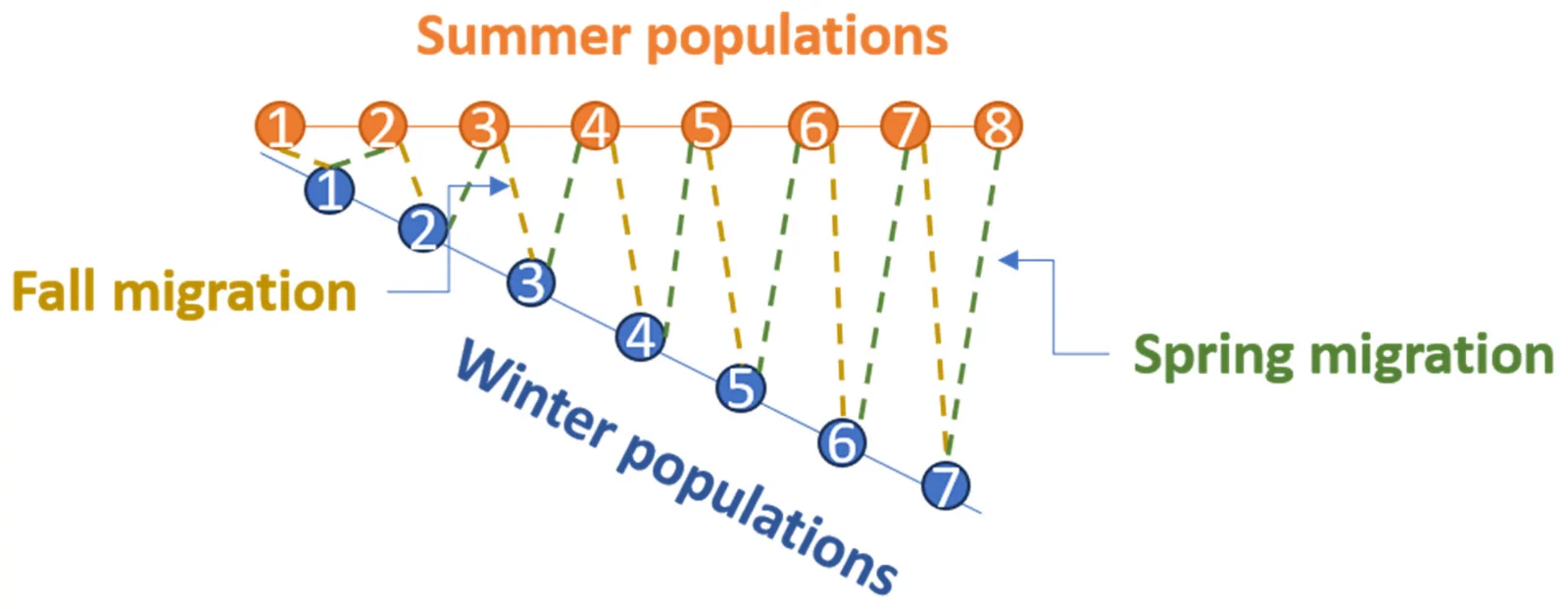
Conceptual illustration of the pattern the migration-mortality hypothesis requires: a stable summer population trend alongside a declining winter population trend. The implicit but unstated consequence of the migration-mortality hypothesis is that reproduction of spring migrants must offset fall migration mortality in equal measure in order to return a declining winter population to summer stationarity.

**Figure 2:**
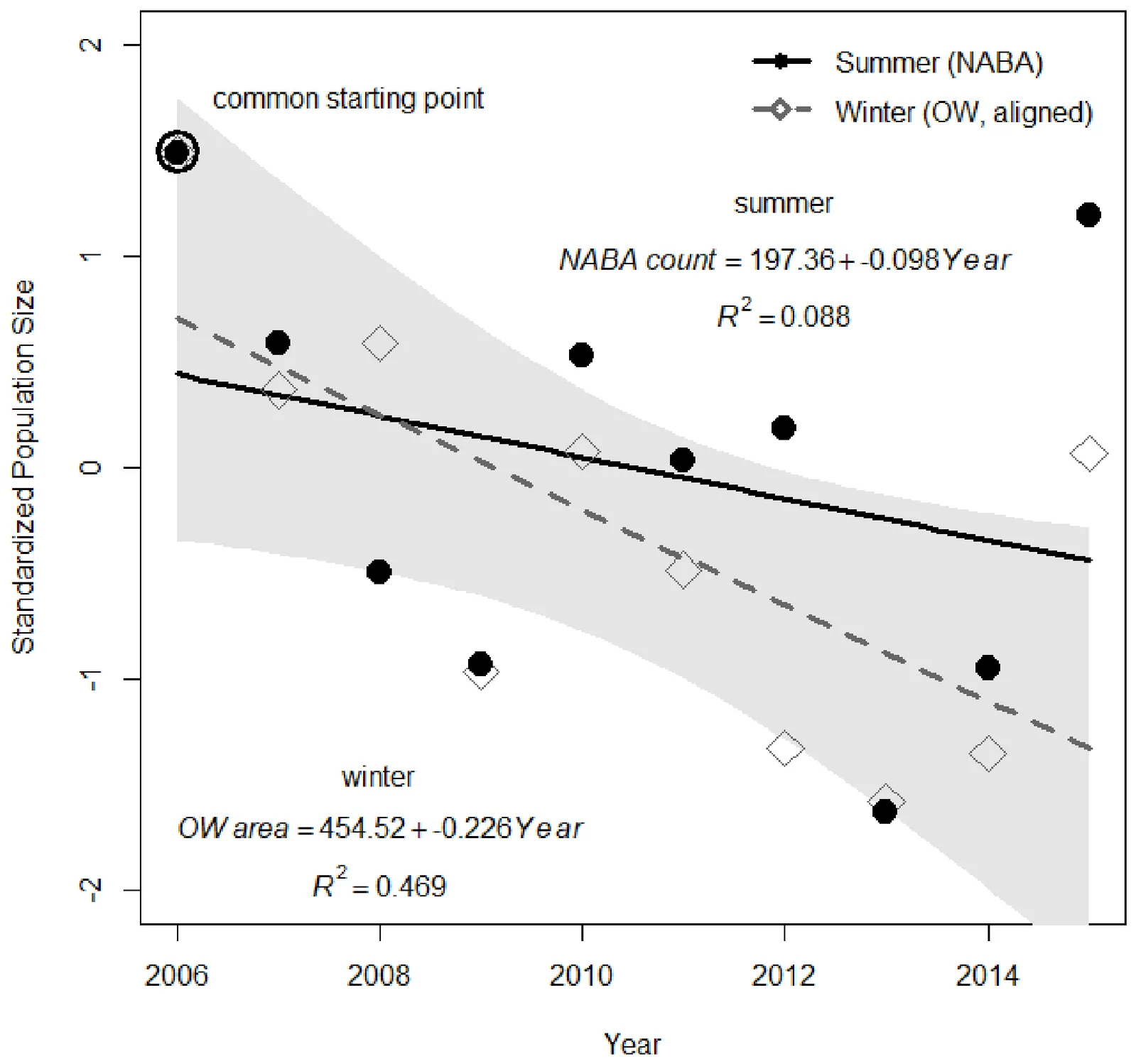
Standardized summer (NABA count, black circles, solid trend line) and winter (overwintering colony area, gray diamonds, dashed trend line and shaded 95% confidence interval) population trends, 2006–2015, aligned to a common starting value in 2006 for visual comparability.

**Figure 3:**
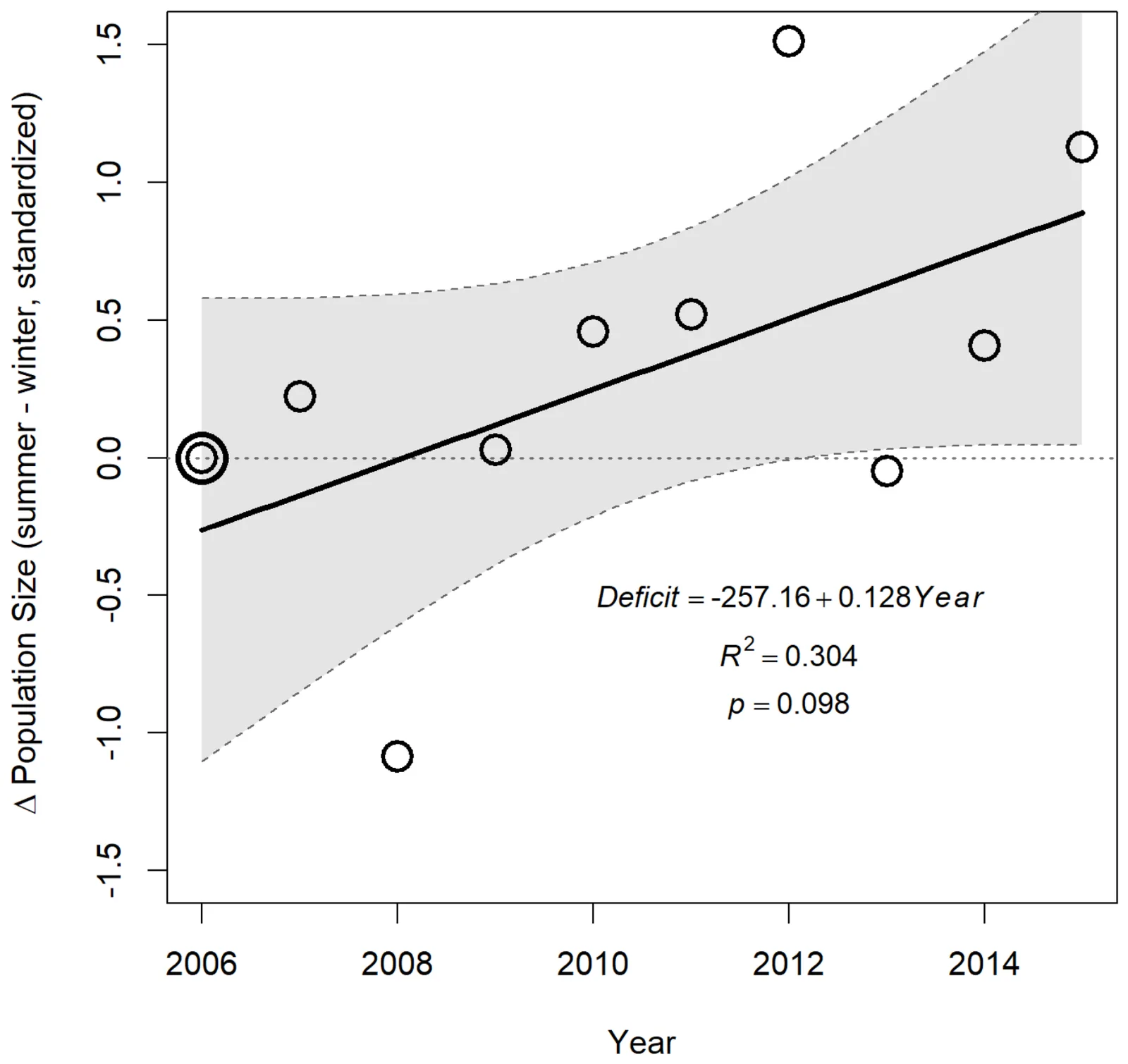
The standardized deficit (*δ*(*t*)) between summer and winter population indices over the same period, with a fitted linear trend and shaded 95% confidence interval. 2006, circled, is the baseline year. The double circle represents the common starting point for summer and winter populations. Under the migration-mortality hypothesis, this deficit represents the reproductive compensation that must be occurring each spring; in the absence of documented compensation, the trend itself is unexplained.

**Figure 4:**
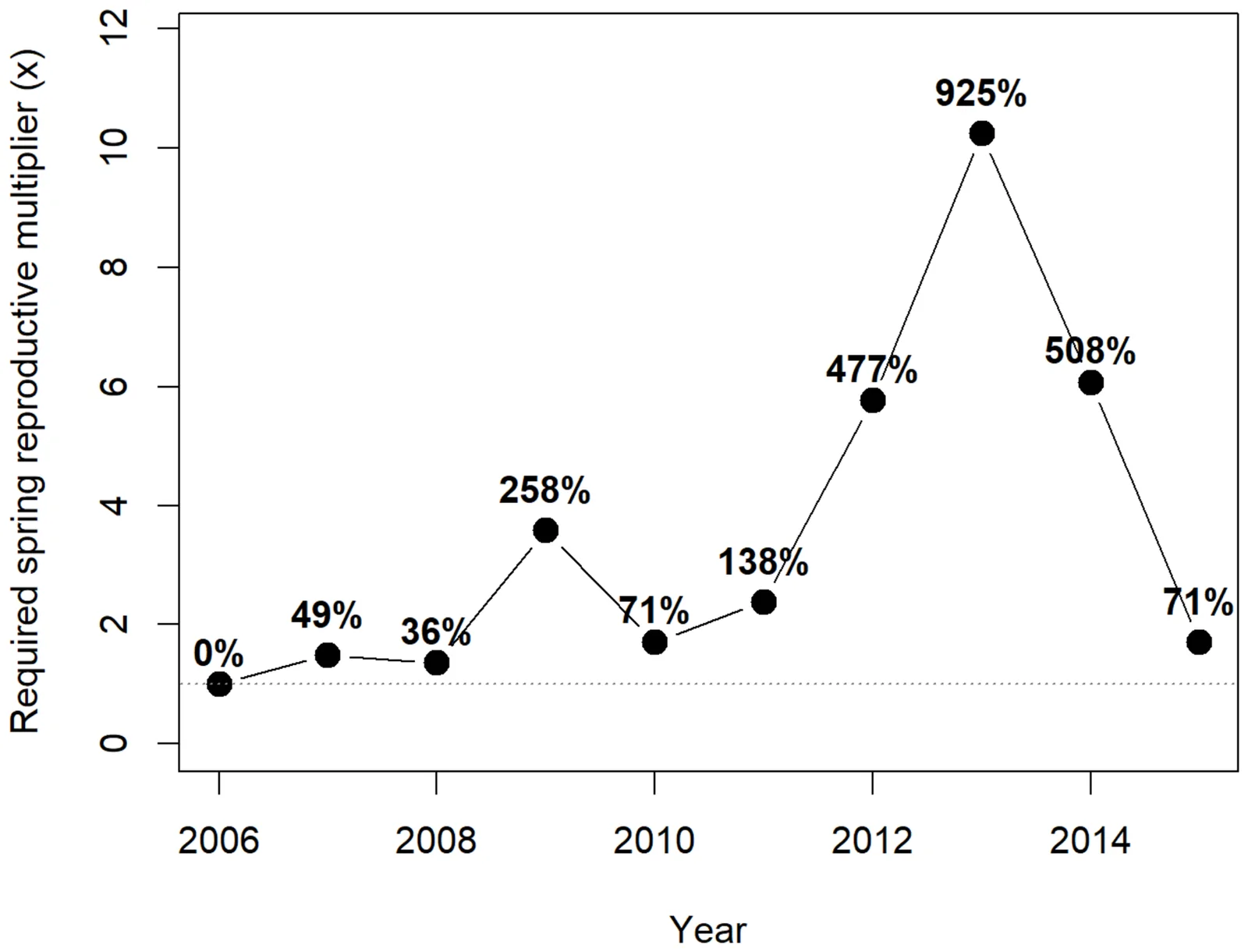
Required increase in spring reproductive output, by year, to restore the overwintering population (OW, hectares) to a stable summer baseline population size. The *y*-axis shows the required reproductive output as a multiplier of the observed value in a given year (e.g., a value of 2 indicates the returning population would need to double); point labels give the same quantity expressed as a percent increase over the observed value (e.g., “930%” for 2013, equivalent to a 10.3-fold increase). The dotted horizontal line at y = 1 indicates no compensation required.

## Data Availability

All data used in this analysis are included in the Supplementary Materials.

## Supplementary Materials

The following supporting information can be downloaded at https://www.mdpi.com/article/10.3390/insects17101089/s1, Figure S1: Standardized summer (NABA) and winter (OW) population trends, 2006–2015, with 95% confidence band on the winter trend. Figure 2 in the manuscript; Figure S2: Signed deficit (summer minus winter, standardized) with fitted linear trend and 95% confidence interval. Figure 3 in the manuscript; Figure S3. Required spring reproductive multiplier by year, labeled with equivalent percent increase over the observed value. Figure 4 in the manuscript.

## Abbreviations

The following abbreviations are used in this manuscript:

NABA	North American Butterfly Association (used for summer population counts)
OW	Overwintering colony area (used for winter population indices)
MLMP	Monarch Larva Monitoring Program (monitoring eggs, larvae, pupae)
IMMP	Integrated Monarch Monitoring Program (standardized surveys)
λ	Lambda, population growth rate (demographic modeling)
R(t)	Spring reproductive output (mathematical notation)
S(t)	Summer breeding-ground population index (mathematical notation)
W(t)	Winter overwintering population index (mathematical notation)
δ(t)	Fall/winter deficit (mathematical notation)
