# Supplementary material for: The Migration-Mortality Hypothesis Requires Reproductive Compensation Not in Evidence
Source: Insects. 2026 Oct 21;17(10):1089. doi: 10.3390/insects17101089 (PMC13704907; doi:10.3390/insects17101089)
Supplement: Supplementary file 1 [file insects-17-01089-s001.zip › insects-4530239-supplementary.pdf]

Supplementary Material: Empirical Analysis Code

The Migration Mortality Hypothesis Requires Reproductive Compensation Not in Evidence

Wayne E Thogmartin, wthogmartin@usgs.gov

Data

Standardized summer (NABA) and winter (overwintering colony area, OW) population indices, 2006-2015. OW is aligned to NABA's 2006 starting value for visual comparability.

```
year <- c(2006, 2007, 2008, 2009, 2010, 2011, 2012, 2013, 2014, 2015)
OW   <- c(6.87, 4.61, 5.06, 1.92, 4.02, 2.89, 1.19, 0.67, 1.13, 4.01)
NABA <- c(7.47, 5.55, 3.22, 2.28, 5.43, 4.36, 4.68, 0.79, 2.25, 6.85)

OW.std   <- as.numeric(scale(OW))
NABA.std <- as.numeric(scale(NABA))
OW.adj   <- OW.std - (OW.std[1] - NABA.std[1])

migrationlimit <- tibble(year, OW, OW.std, OW.adj, NABA, NABA.std)
migrationlimit
```

```
## # A tibble: 10 x 6
##     year    OW OW.std  OW.adj  NABA NABA.std
##    <dbl> <dbl>  <dbl>   <dbl> <dbl>    <dbl>
##  1  2006  6.87  1.80    1.49    7.47   1.49
##  2  2007  4.61  0.680   0.366   5.55   0.589
##  3  2008  5.06  0.903   0.589   3.22  -0.499
##  4  2009  1.92 -0.652  -0.966   2.28  -0.937
##  5  2010  4.02  0.388   0.0738  5.43   0.533
##  6  2011  2.89 -0.172  -0.486   4.36   0.0336
##  7  2012  1.19 -1.01   -1.33    4.68   0.183
##  8  2013  0.67 -1.27   -1.59    0.79  -1.63
##  9  2014  1.13 -1.04   -1.36    2.25  -0.951
## 10  2015  4.01  0.383   0.0688  6.85   1.20
```

Trend Models

Separate linear trends for summer (NABA), winter (OW, aligned), and the signed deficit between them (summer minus winter), per Section 3's formalization $R(t) \approx S_0 - W(t)$.

```
yb.NABA <- lm(NABA.std ~ year, data = migrationlimit)
yw.OW   <- lm(OW.adj ~ year, data = migrationlimit)

deficit_df <- data.frame(year = migrationlimit$year,
                         deficit = migrationlimit$NABA.std - migrationlimit$OW.adj)
y.recovered <- lm(deficit ~ year, data = deficit_df)
```

```r
summary(yb.NABA)
```

```
##
## Call:
## lm(formula = NABA.std ~ year, data = migrationlimit)
##
## Residuals:
##     Min      1Q  Median      3Q     Max
## -1.3874 -0.7099  0.1641  0.4455  1.6376
##
## Coefficients:
##              Estimate Std. Error t value Pr(>|t|)
## (Intercept) 197.36053  224.16710    0.88    0.404
## year         -0.09816    0.11150   -0.88    0.404
##
## Residual standard error: 1.013 on 8 degrees of freedom
## Multiple R-squared:  0.08833,    Adjusted R-squared:  -0.02563
## F-statistic: 0.7751 on 1 and 8 DF,  p-value: 0.4043
```

```r
summary(yw.OW)
```

```
##
## Call:
## lm(formula = OW.adj ~ year, data = migrationlimit)
##
## Residuals:
##      Min       1Q   Median       3Q      Max
## -0.99161 -0.56877 -0.08528  0.32164  1.40088
##
## Coefficients:
##              Estimate Std. Error t value Pr(>|t|)
## (Intercept) 454.52252  171.05617   2.657   0.0289 *
## year         -0.22623    0.08508  -2.659   0.0289 *
## ---
## Signif. codes:  0 '***' 0.001 '**' 0.01 '*' 0.05 '.' 0.1 ' ' 1
##
## Residual standard error: 0.7728 on 8 degrees of freedom
## Multiple R-squared:  0.4692, Adjusted R-squared:  0.4028
## F-statistic:  7.07 on 1 and 8 DF,  p-value: 0.02885
```

```r
summary(y.recovered)
```

```
##
## Call:
## lm(formula = deficit ~ year, data = deficit_df)
##
## Residuals:
##     Min      1Q  Median      3Q     Max
## -1.0812 -0.2902  0.1754  0.2559  1.0047
##
## Coefficients:
```

```
##                Estimate Std. Error t value Pr(>|t|)
## (Intercept) -257.16199  137.67134  -1.868   0.0987 .
## year           0.12807    0.06848   1.870   0.0984 .
## ---
## Signif. codes:  0 '***' 0.001 '**' 0.01 '*' 0.05 '.' 0.1 ' ' 1
##
## Residual standard error: 0.622 on 8 degrees of freedom
## Multiple R-squared:  0.3042, Adjusted R-squared:  0.2172
## F-statistic: 3.498 on 1 and 8 DF,  p-value: 0.09837
```

Code for **Figure 2: Summer vs. Winter Population Trends**

```
newx <- seq(2006, 2015, length.out = 50)
preds.OW <- predict(yw.OW, newdata = data.frame(year = newx), interval = "confidence")

plot(NABA.std ~ year, data = migrationlimit,
     xlim = c(2006, 2015), ylim = c(-2, 2), cex = 2, pch = 19, col = "black",
     xlab = "Year", ylab = "Standardized Population Size"#,
     #main = "Summer vs. Winter Population Trends (2006-2015)"
     )

polygon(c(rev(newx), newx), c(rev(preds.OW[, 3]), preds.OW[, 2]), col = "gray90", border = NA)
curve(yb.NABA$coef[1] + yb.NABA$coef[2] * x, col = "black", add = TRUE, lwd = 3)
lines(newx, preds.OW[, 1], col = "gray40", lwd = 3, lty = 2)
points(OW.adj ~ year, data = migrationlimit, cex = 2, pch = 5, col = "gray40")
points(NABA.std ~ year, data = migrationlimit, cex = 2, pch = 19, col = "black")

points(y = 1.5, x = 2006, cex = 3.25, lwd = 3, pch = 1, col = "black")
text(y = 1.75, x = 2007.7, label = "common starting point")
text(y = -1.45, x = 2008, label = "winter")
text(y = -1.70, x = 2008, eqn.OW)
text(y = -1.95, x = 2008, r2.OW)
text(y = 1.25, x = 2012, label = "summer")
text(y = 1.00, x = 2012, eqn.NABA)
text(y = 0.75, x = 2012, r2.NABA)

legend("topright", legend = c("Summer (NABA)", "Winter (OW, aligned)"),
       col = c("black", "gray40"), lty = c(1, 2), lwd = 3, pch = c(19,5), bty = "n")
```

Code for **Figure 3: De icit Between Summer and Winter Populations**

```
newx <- seq(2006, 2015, length.out = 50)
preds <- predict(y.recovered, newdata = data.frame(year = newx), interval = "confidence")

plot(deficit_df$year, deficit_df$deficit, type = "n",
     xlab = "Year", xlim = c(2006, 2015), ylim = c(-1.5, 1.5),
     ylab = expression(paste(Delta, " Population Size (summer - winter, standardized)")))

abline(h = 0, lty = 3, col = "gray50", lwd = 1.5)
```

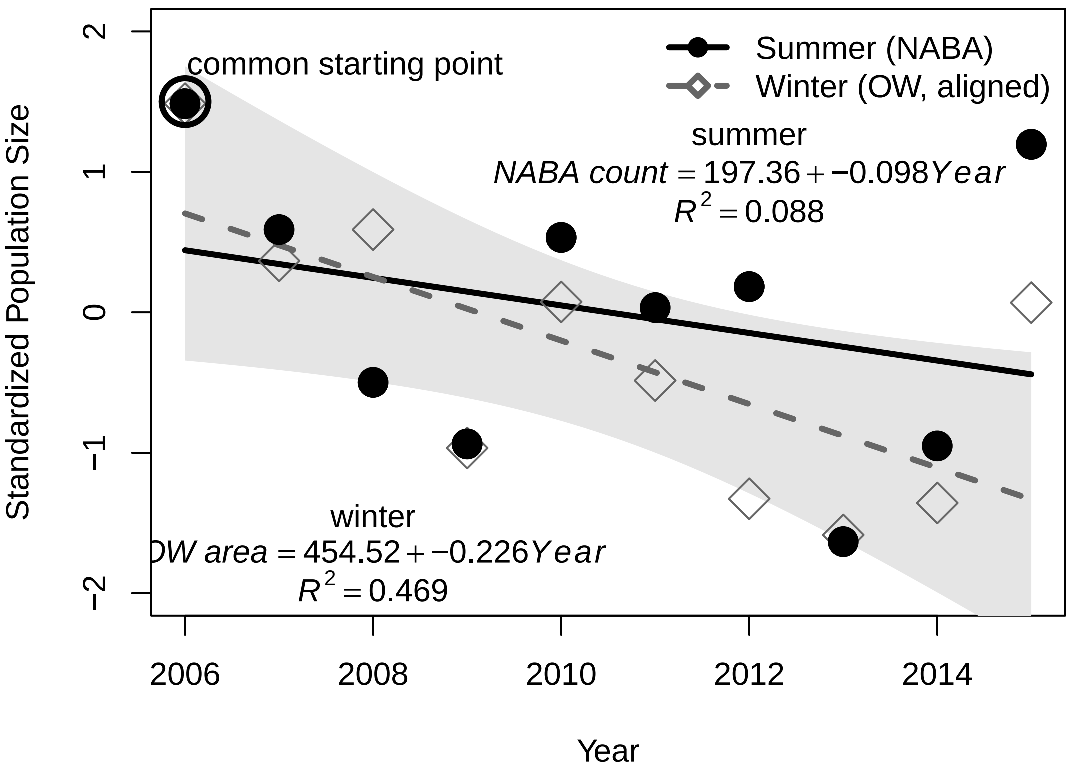

Figure S1: Standardized summer (NABA) and winter (OW) population trends, 2006-2015, with 95% confidence band on the winter trend. Figure 2 in the manuscript.

```
polygon(c(rev(newx), newx), c(rev(preds[, 3]), preds[, 2]), col = rgb(0, 0, 0, 0.1), border = NA)
lines(newx, preds[, 3], lty = "dashed", col = "gray40")
lines(newx, preds[, 2], lty = "dashed", col = "gray40")
lines(newx, preds[, 1], col = "black", lwd = 3)
points(deficit_df$year, deficit_df$deficit, pch = 1, cex = 2, lwd = 2, col = "black")
points(y = deficit_df$deficit[1], x = 2006, cex = 3.25, lwd = 3, pch = 1, col = "black")

text(y = -0.5, x = 2012, eqn.recov, cex = 0.9)
text(y = -0.7, x = 2012, r2.recov, cex = 0.9)
text(y = -0.9, x = 2012, p.recov, cex = 0.9)
```

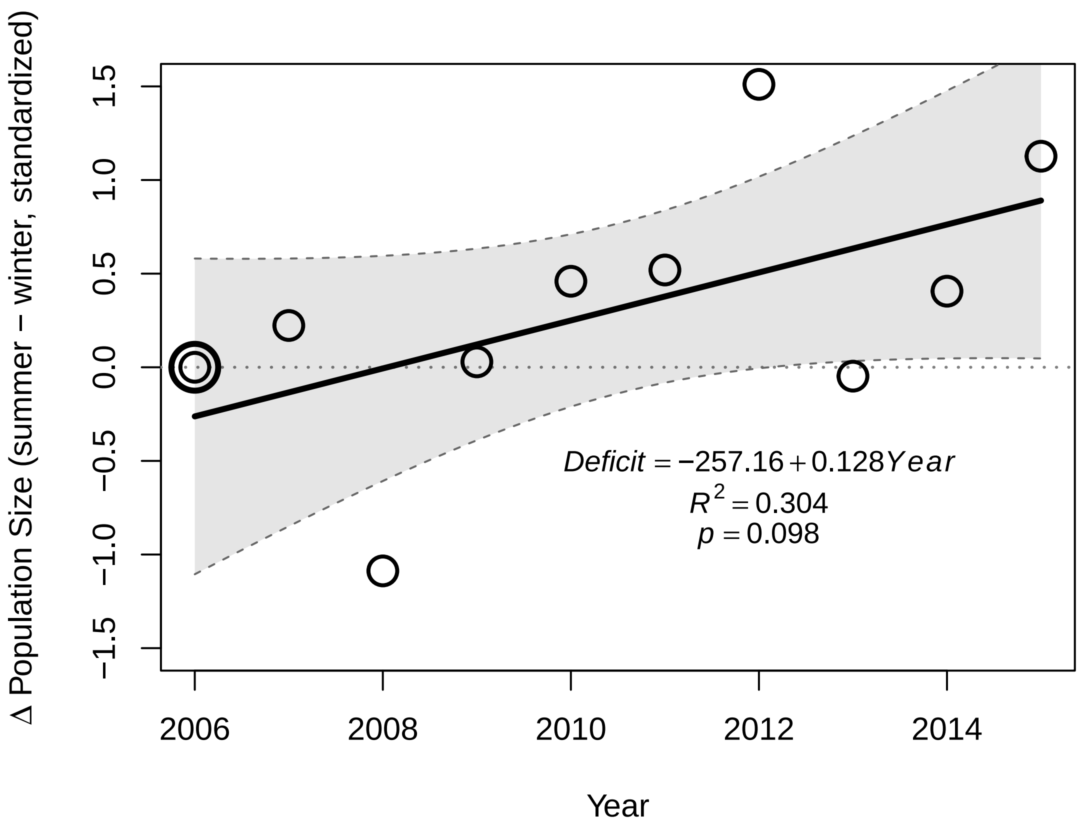

Figure S2: Signed deficit (summer minus winter, standardized) with fitted linear trend and 95% confidence interval. Figure 3 in the manuscript.

Sensitivity Check: Alternative Alignment Method

Does the deficit trend hold under mean-based alignment of OW instead of anchor-year (2006) alignment?

```
OW.adj.meanalign <- OW.std - (mean(OW.std) - mean(NABA.std))
y.recovered.meanalign <- lm(NABA.std - OW.adj.meanalign ~ migrationlimit$year)
```

```r
cat("Anchor-year alignment (2006):\n")
```

```
## Anchor-year alignment (2006):
```

```r
print(summary(y.recovered)$coefficients)
```

```
##                Estimate  Std. Error   t value   Pr(>|t|)
## (Intercept) -257.1619950 137.6713386 -1.867941 0.09871883
## year           0.1280657   0.0684761  1.870224 0.09837310
```

```r
cat("\nMean alignment (whole series):\n")
```

```
##
## Mean alignment (whole series):
```

```r
print(summary(y.recovered.meanalign)$coefficients)
```

```
##                          Estimate  Std. Error   t value   Pr(>|t|)
## (Intercept)          -257.4760001 137.6713386 -1.870222 0.09837339
## migrationlimit$year     0.1280657   0.0684761  1.870224 0.09837310
```

Required Compensation Magnitude

Required percent increase and multiplier in spring reproductive output needed to restore each year's overwintering population (OW, hectares) to the 2006 baseline. Benchmarked against Oberhauser et al. (2017, *Ecological Entomology* 42:51-60, Table 2): ~2% reflects their most effective single-region structural scenario; these are sustained, multi-region vital-rate improvements, not single-season output, so the comparison is conservative.

```r
W0 <- OW[1]
required_pct_fixed  <- ((W0 - OW) / OW) * 100
required_multiplier <- W0 / OW

compensation_table <- tibble(
  year = year,
  OW_ha = OW,
  required_pct_increase = round(required_pct_fixed, 1),
  required_multiplier_x = round(required_multiplier, 2)
)

oberhauser_best_single_region_pct <- 2  # Table 2, scenarios 6 & 7
compensation_table$fold_vs_best_single_region <-
  round(compensation_table$required_pct_increase / oberhauser_best_single_region_pct, 0)

compensation_table
```

```
## # A tibble: 10 x 5
##     year OW_ha required_pct_increase required_multiplier_x
##    <dbl> <dbl>                 <dbl>                 <dbl>
```

```
## 1  2006  6.87                  0                    1
## 2  2007  4.61                 49                    1.49
## 3  2008  5.06                 35.8                  1.36
## 4  2009  1.92                258.                   3.58
## 5  2010  4.02                 70.9                  1.71
## 6  2011  2.89                138.                   2.38
## 7  2012  1.19                477.                   5.77
## 8  2013  0.67                925.                  10.2
## 9  2014  1.13                508                    6.08
## 10 2015  4.01                 71.3                  1.71
## # i 1 more variable: fold_vs_best_single_region <dbl>
```

Code for **Figure 4: Required Spring Reproductive Multiplier by Year**

```
ymax <- max(compensation_table$required_multiplier_x) * 1.15
plot(compensation_table$year, compensation_table$required_multiplier_x,
     type = "b", pch = 19, cex = 1.5,
     xlab = "Year", ylab = "Required spring reproductive multiplier (x)",
     ylim = c(0, ymax))
abline(h = 1, lty = 3, col = "gray50")

label_offset <- ifelse(seq_along(year) %% 2 == 0, 0.7, 0.55)
text(x = compensation_table$year,
     y = compensation_table$required_multiplier_x + label_offset,
     labels = paste0(round(compensation_table$required_pct_increase, 0), "%"),
     cex = 1, col = "black", font = 2)
```

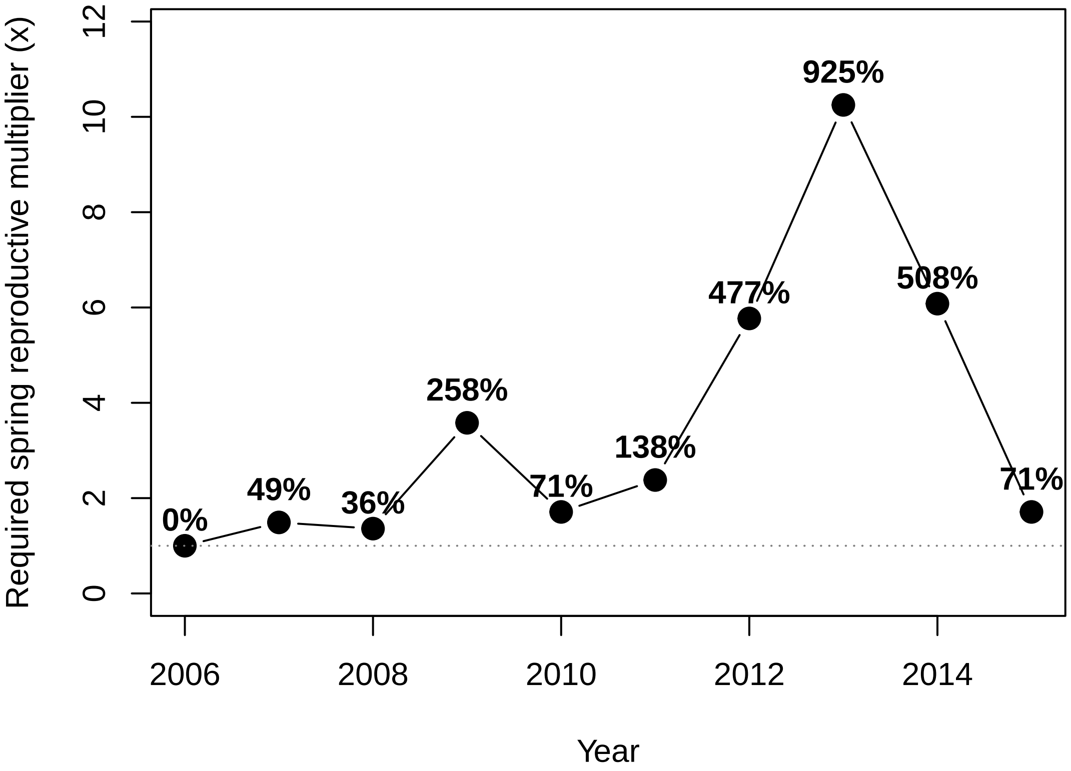

Figure S3: Required spring reproductive multiplier by year, labeled with equivalent percent increase over the observed value. Figure 4 in the manuscript.
